# Azo-Linkage Redox Metal–Organic Framework Incorporating Carbon Nanotubes for High-Performance Aqueous Energy Storage

**DOI:** 10.3390/molecules28227479

**Published:** 2023-11-08

**Authors:** Hualei Zhang, Xinlei Wang, Jie Zhou, Weihua Tang

**Affiliations:** 1 College of Materials, Xiamen University, Xiamen 361005, China; 2 School of Chemistry and Chemical Engineering, Nanjing University of Science and Technology, Nanjing 210094, China

**Keywords:** metal–organic frameworks, post-synthetic modification, redox pendants, film electrode, energy storage

## Abstract

The design of well-defined hierarchical free-standing electrodes for robust high-performance energy storage is challenging. We report herein that azo-linkage redox metal–organic frameworks (MOFs) incorporate single-walled carbon nanotubes (CNTs) as flexible electrodes. The in situ-guided growth, crystallinity and morphology of UiO-66-NO_2_ MOFs were finely controlled in the presence of CNTs. The MOFs’ covalent anchoring to CNTs and solvothermal grafting anthraquinone (AQ) pendants endow the hybrid (denoted as CNT@UiO-66-AQ) with greatly improved conductivity, charge storage pathways and electrochemical dynamics. The flexible CNT@UiO-66-AQ displays a highest areal specific capacitance of 302.3 mF cm^−2^ (at 1 mA cm^−2^) in −0.4~0.9 V potential window, together with 100% capacitance retention over 5000 cycles at 5 mA cm^−2^. Its assembled symmetrical supercapacitor (SSC) achieves a maximum energy density of 0.037 mWh cm^−2^ and a maximum power density of 10.4 mW cm^−2^, outperforming many MOFs-hybrids-based SSCs in the literature. Our work may open a new avenue for preparing azo-coupled redox MOFs hybrids with carbaneous substrates for high-performance robust aqueous energy storage.

## 1. Introduction

Metal-organic frameworks (MOFs) have flourished as a fascinating class of electrochemical energy storage electrode materials [1,2,3,4,5,6,7,8]. Featuring porous crystalline structures, they are composed of metal cluster nodes and organic linkers/ligands. Their diversified functionalities, unique morphologies and tunable porosities endow them with superior charge storage capability. However, the low conductivity of pristine MOFs is acknowledged as the dominant limitation for their practical applications. One facile strategy to address this issue is to incorporate conductive components (e.g., carbon-based materials and conducting polymers) into MOFs skeletons, affording improved conductivity and possible extra charge storage capacity [9,10,11,12,13,14]. Furthermore, the functional surface of carbon materials can guide the uniform growth of MOFs along them and stabilize the as-formed hierarchical structures [15,16,17]. For example, ultrathin nickel MOF-based two-dimensional (2D) nanosheets were prepared with finely tuned thickness and specific surface with the aid of in situ interpenetration of carboxylated carbon nanotubes (CNTs) [16]. The 2D integrated hybrid electrode delivered a superior specific capacity of 680 C g^−1^ at 1 A g^−1^ with 68% capacity retention at 10 A g^−1^.

Besides considering conductivity, it is crucial to improve MOFs’ capacity by introducing new conjugated ligands or redox functionalities. The incorporation of redox ligands or pendants in/onto MOFs skeletons efficiently improves electrochemical charge storage through reversible redox reactions in aqueous electrolytes [18,19,20]. In accordance with this, Chen’s group recently reported a two-dimensional copper–benzoquinoid MOF to serve as lithium-ion battery cathode [21]. The hybrid electrode delivered a high reversible capacity (387 mAh g^−1^) and good cycling stability, where a three-electron redox reaction for each coordination unit was further unveiled using comprehensive spectroscopic techniques. However, for redox ligand-based MOFs, the change in the oxidation state of ligands may impact the coordination geometry of metal nodes. This creates great challenges in selecting suitable building blocks for stabilizing the structure under electrochemical conditions. 

On the other hand, the grafting of functional pendants via post-synthetic modification has matured into an efficient and facile strategy to broaden the scope of MOFs [22,23]. The diverse functional groups (e.g., -NH_2_, -OH, -SH, et al.) on ligands afford great possibilities for chemical modification to generate multi-purpose structures. Meanwhile, the covalent bonding can further prevent the electrodes from dissolving into electrolytes. Post-synthetic coupling of redox pendants can endow MOFs with redox activity to improve their charge storage capacity and extra ion transfer to promote efficient electrochemical dynamics. For instance, quinones and their derivatives, showcasing reversible redox reactions and high theoretical capacity, have been successfully explored for batteries and capacitors [24,25,26,27]. However, the post-synthetic grafting of MOFs using redox quinones has rarely been reported, which is mainly attributed to a lack of reliable synthetic approaches for linkage building. Zirconium-based MOFs are most commonly used due to the availability of a large variety of benzene dicarboxylic acids for ligands, and their high chemical stability can endure further chemical modifications. 

**Figure 1:**
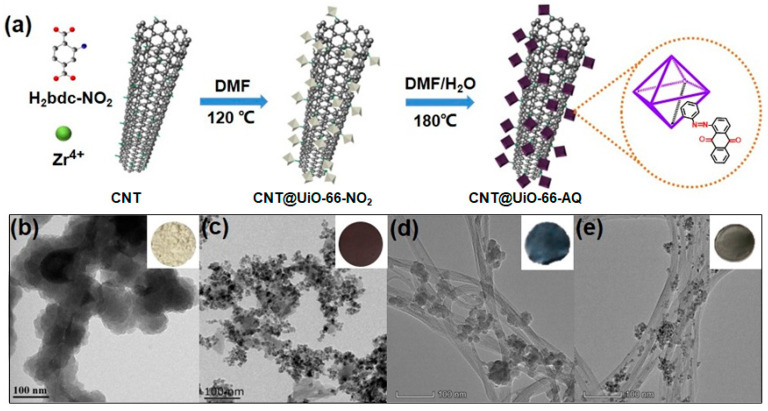
(**a**) Schematic route of the post-synthetic grafting AQ pendants onto MOFs with in situ growth guided by carboxylated single-walled CNTs. TEM images of (**b**) UiO-66-NO_2_, (**c**) UiO-66-AQ, (**d**) CNT@UiO-66-NO_2_, and (**e**) CNT@UiO-66-AQ. The insets of (**b**–**e**) show the photos of the corresponding filter cakes.

In this work, we report the covalent bonding of redox pendants onto MOFs via a facile post-synthetic approach and their incorporation with CNTs as hybrid self-standing electrodes for high-performance and robust energy storage in aqueous electrolytes (Figure 1). The stable azo-coupled MOF was readily achieved via a one-pot solvothermal reaction between 1-nitroanthraquinone and nitro-substituted UiO-66 (denoted as UiO-66-NO_2_) in a mixture solution of dimethyl formamide and water. More importantly, we further incorporate carboxylated carbon nanotubes CNTs for the in-situ growth of MOFs to fine tune their crystallinity and morphology as well as their conductivity. The as-obtained redox MOFs hybrid (denoted as CNTs@UiO-66-AQ) with CNTs features azo-linked anthraquinone (AQ) pendants and well-aligned CNTs as the charge transfer channels, where capacitance and redox reaction contribute to significantly improved charge storage. Consequently, the self-standing CNT@UiO-66-AQ electrode delivers a maximum areal specific capacitance of 302.3 mF cm^−2^ at 1 mA cm^−2^ in 1 M H_2_SO_4_ electrolyte. Excellent rate performance is revealed by the undecayed capacitance after 5000 cycles at 5 mA cm^−2^. Importantly, two film electrodes-assembled symmetrical supercapacitors (SSCs) exhibit a maximum energy density of 0.037 mWh cm^−2^ and a highest power density of 10.4 mW cm^−2^, outperforming most MOFs-based SSCs in the literature.

## 2. Results and Discussion

**Figure 2:**
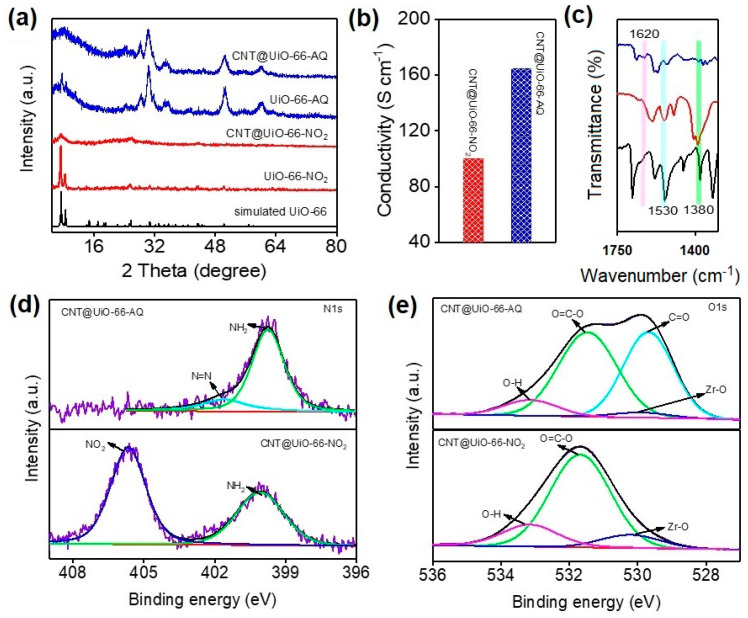
(**a**) XRD patterns of as-synthesized MOFs and their hybrids with CNTs. (**b**) Conductivity of MOF hybrids with CNTs. (**c**) FT-IR spectra of 1-nitroanthraquinone (bottom curve), UiO-66-NO_2_ (middle curve) and UiO-66-AQ (top curve). High-resolution XPS spectra for (**d**) N 1s and (**e**) O 1s regions as well as their peak deconvolution (color lines) in CNT@UiO-66-AQ and CNT@UiO-66-NO_2_.

As depicted in Figure 1a, UiO-66-NO_2_ MOF is steadily anchored onto the CNT surface via coordinative interactions. The post-synthetic solvothermal reaction converts two nitro groups into robust azo-linkage between AQ pendants and UiO-66 skeletons. The modification of redox quinones extended the potential window and enhanced the charge storage. The CNT incorporated redox MOF hybrids (CNT@UiO-66-AQ) can be easily prepared using two-step solvothermal reactions (Figure 1a). The direct azo-coupling redox AQ pendants onto MOF skeletons afforded a darker-colored product, implying successful chemical modifications. X-ray diffraction (XRD) spectroscopy was used to reveal the evolution of crystal structures of the MOFs accompanying the post-synthetic functionalization. As depicted in Figure 2a, UiO-66-NO_2_ exhibits peak patterns consistent with the simulated results for UiO-66, indicating that the nitro substituents have no effect on the crystalline structure of the MOF skeleton. After the azo-coupling AQ was added to the Zr-MOF skeleton, UiO-66-AQ still exhibit the typical crystalline peaks despite the decreased intensities compared with UiO-66-NO_2_, confirming the unchanged framework structures [28]. Importantly, new strong peaks appear in the region of 28°~36°, 50° and 60°, which are attributed to the packing and stacking of AQ pendants within MOF holes. It should be noted that the incorporation of CNTs results in overlapping and broadening of MOFs’ peaks, indicating a certain decrease in the crystallinity of MOFs. However, with the grafting of redox pendants onto the electron-donating MOF skeleton, the resulting intermolecular charge transfer and packing pendants induced charge hopping that will significantly improve the charge transport in CNT@UiO-66-AQ hybrid [27]. The four-point probe measurement shows CNT@UiO-66-AQ hybrid exhibits nearly 62% improved conductivity (~164.7 S cm^−1^) than CNT@UiO-66-NO_2_ (100.1 S cm^−1^) (Figure 2b). Fourier transform infrared spectroscopy (FT-IR) was applied to confirm the successful azo-coupling of AQ pendants. As demonstrated in Figure 2c and Figure S1, the characteristic vibration of -NO_2_ can be observed at peak 1530 and 1380 cm^−1^ for 1-nitroanthraquinone and UiO-66-NO_2_, respectively [29]. Such peak assigned to -NO_2_ vibration disappears; instead, UiO-66-AQ exhibits a new peak at 1620 cm^−1^, representing the azo group [30]. This suggests the successful conversion of nitro into the azo group in our post-synthetic modification. However, the nitrogen-containing functionality change is difficult to detect within the FT-IR spectra of CNT@UiO-66-NO_2_ and CNT@UiO-66-AQ (Figure S1, Supplementary Materials), which can be attributed to the blending of CNTs. 

The formation of azo linkage is clearly confirmed with X-ray photoelectron spectroscopy (XPS). The N 1s spectrum of CNT@UiO-66-NO_2_ shows two obvious peaks at 405.6 and 400.0 eV, corresponding to -NO_2_ and -NH_2_, respectively (Figure 2d). The -NH_2_ peaks originate from 3-aminobenzoic acid for UiO-66 [31]. After nitro–azo conversion, the characteristic peak of -NO_2_ from UiO-66-NO_2_ vanishes, and a new peak at 399.1 eV assigned to the azo group appears for CNT@UiO-66-AQ [32]. At the same time, CNT@UiO-66-AQ displays the peaks for C=O at 529.7 and 287.1 eV in the high-resolution O 1s and C 1s spectra, respectively (Figure 2e and Figure S2a, Supplementary Materials). This further confirms the covalent bonding of AQ pendants onto the UiO-66-based MOF skeleton [33,34]. As expected, both CNT@UiO-66-NO_2_ and CNT@UiO-66-AQ exhibit the same peaks at 185.2 and 182.6 eV for Zr 3d spectra, indicating the unchanged charge state of Zr during nitro–azo conversion (Figure S2b, Supplementary Materials) [35]. Similar phenomena are observed for the evolution of four elements (ca. C 1s, N 1s, O 1s and Zr 3d) valences accompanying UiO-66-NO_2_ transform into UiO-66-AQ (Figure S3, Supplementary Materials). 

**Figure 3:**
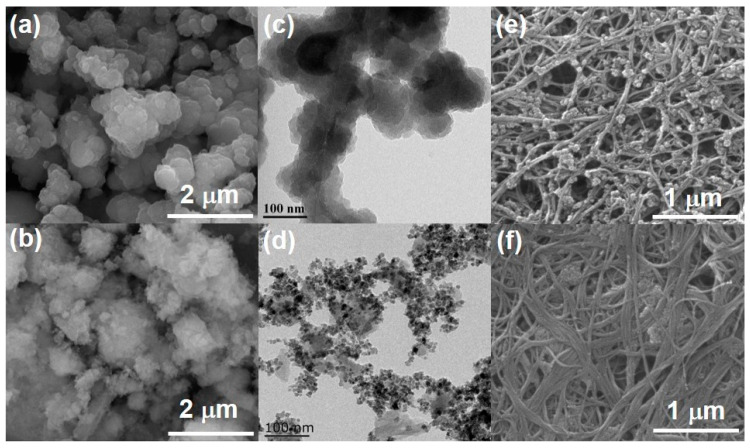
SEM images of (**a**) UiO-66-NO_2_, (**b**) UiO-66-AQ, (**e**) CNT@UiO-66-NO_2_ and (**f**) CNT@UiO-66-AQ. TEM images of (**c**) UiO-66-NO_2_ and (**d**) UiO-66-AQ.

The morphology of MOFs and their CNTs hybrids is assessed using scanning electron microscopy (SEM) and a transmission electron microscope (TEM). UiO-66-NO_2_ exhibits heavily aggregated particles with irregular shapes, which are ~1 μm in diameter (Figure 3a). This coincides with the TEM image (Figure 3c), wherein low transmittance is observed for large stacked particles. After azo-coupling AQ, UiO-66-AQ takes the form of soft and fluffy cottons (Figure 3b), and nanosized particles can be observed in the TEM image (Figure 3d). This can explain the decreased peak intensity in XRD patterns for UiO-66-AQ due to its loosely packed particle structure. It should be noted that UiO-66-NO_2_ and UiO-66-AQ present almost the same homogeneous and fine distribution of four elements (including C, Zr, N and O), as shown in Figure S4 (Supplementary Materials). The guided growth of MOFs along 2D CNTs is demonstrated in Figure 3e,f. UiO-66-NO_2_ nanoparticles (upto ~100 nm in diameter) homogeneously attach to CNTs to construct a porous hierarchical hybrid. Even smaller-sized UiO-66-AQ nanoparticles fill the cavities of well-aligned CNTs. 

**Figure 4:**
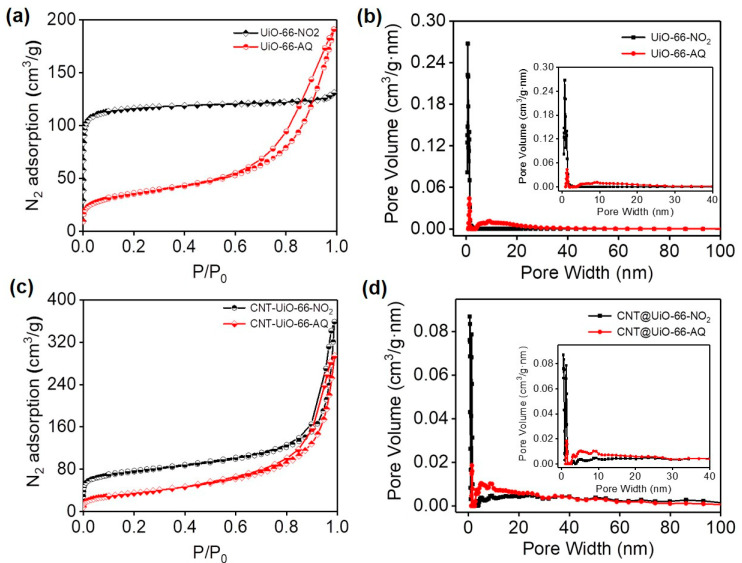
N_2_ adsorption and desorption isotherms for (**a**) UiO-66-NO_2_ and UiO-66-AQ and (**c**) CNT@UiO-66-NO_2_ and CNT@UiO-66-AQ, and pore size distributions for (**b**) UiO-66-NO_2_ and UiO-66-AQ and (**d**) CNT@UiO-66-NO_2_ and CNT@UiO-66-AQ.

The porous structures of UiO-66 derived MOFs and their growth-guided CNTs hybrids were further studied using nitrogen adsorption–desorption analysis. As depicted in Figure 4, UiO-66-NO_2_ exhibits a typical type-I isotherm for nitrogen adsorption, revealing a specific surface area of 462.6 m^2^ g^−1^. The AQ-bonded UiO-66-AQ behaves similarly to a type-Ⅳ isotherm to afford a reduced surface area of 129.2 m^2^ g^−1^. However, UiO-66-AQ shows significantly increased pore sizes (from 1.7 to 9.1 nm) and pore volume (from 0.2 to 0.3 cm^3^g^−1^) in comparison to UiO-66-NO_2_. These results indicate the azo-coupled AQ modification can greatly relieve the stacking of UiO-66 based MOFs, which agrees well with the morphology study in Figure 3.

In terms of porosity investigation, both CNT@UiO-66-NO_2_ and CNT@UiO-66-AQ display typical type-Ⅳ isotherms (Figure 4c). CNT@UiO-66-NO_2_ showcases a specific surface area (274.5 m^2^ g^−1^) larger than CNT@UiO-66-AQ (127.1 m^2^ g^−1^) but lower than UiO-66-NO_2_. Correspondingly, the pore size is increased from 8.1 nm for CNT@UiO-66-NO_2_ to 14.3 nm for CNT@UiO-66-AQ (Figure 4d). Such microporous and mesoporous structures of our as-developed redox MOFs and CNT hybrid films can offer sufficient low-resistance channels for electrolyte penetration when used as electrodes [36]. Besides increased pore size and volume, the azo-coupling post-synthetic modification also leads to improved thermal stability for UiO-66-AQ and CNT@UiO-66-AQ compared to their nitro counterparts, as indicated by the thermogravimetric analysis (TGA) in Figure S6 (Supplementary Materials).

**Figure 5:**
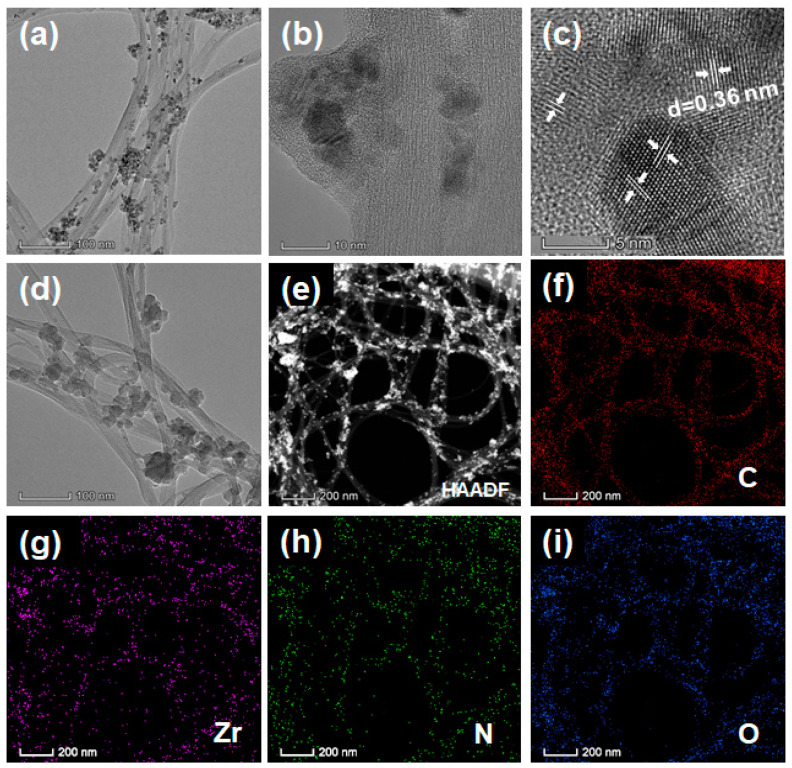
(**a**) TEM and (**b**,**c**) HRTEM images CNT@UiO-66-AQ. (**d**) TEM image of CNT@UiO-66-NO_2_. (**e**) HAADF image of CNT@UiO-66-AQ and (**f**–**i**) corresponding element mapping of C, Zr, N and O, respectively.

The coordinative effect of the carboxyl group toward Zr ions renders guided and uniform distribution of MOF crystals along CNT nanofibers for CNT@UiO-66-NO_2_ (Figure 3e), which can be more clearly observed in the TEM image (Figure 5). The heteronuclear and crystal growth of UiO-66-NO_2_ MOFs on CNTs is thus concluded [35]. As a comparison, CNT@UiO-66-AQ shows a smoother surface with several neighboring CNTs aligning together into a nano-belt and MOFs filled within the cavities (Figure 3f). Such morphology can be clearly observed via high-resolution TEM (HRTEM) (Figure 5a,b). CNT@UiO-66-AQ nanoparticles upto ~10 nm in diameter tightly attach to CNTs’ surfaces after grafting AQ pendants. Interestingly, the HRTEM images demonstrate clear crystal lattice fringes for CNT@UiO-66-AQ with a *d*-spacing of 0.36 nm (Figure 5b,c). However, such a phenomenon cannot be observed for CNT@UiO-66-NO_2_ (Figure S5, Supplementary Materials), indicating the restacking of organic pendants. Nonetheless, typical high-angle annular dark field (HAADF) images reveal the uniform distribution of C, N, O, and Zr for both samples (Figure 5e–i and Figure S5c, Supplementary Materials).

The electrochemical performance of a redox MOFs hybrid with CNTs was first evaluated in a three-electrode cell with 1 M H_2_SO_4_ electrolyte. Figure S7a (Supplementary Materials) shows the typical CV curves of UiO-66-NO_2_ and UiO-66-AQ electrodes at a scan rate of 30 mV s^−1^. In comparison to UiO-66-NO_2_, UiO-66-AQ shows a larger area under CV curve and a pair of redox peaks in the region of −0.3~−0.1 V, corresponding to the proton-coupled faradaic reactions of carbonyl groups. UiO-66-AQ also displays a wider potential window (−0.4~1 V vs. 0~0.8 V) and longer charge/discharge time (50 s vs. 220 s), as depicted by the galvanostatic charge–discharge (GCD) curves in Figure S7b (Supplementary Materials). Based on the discharge curve, UiO-66-AQ presents a 4-fold specific capacitance (40 F g^−1^) compared to UiO-66-NO_2_ (10 F g^−1^) at a current density of 0.5 A g^−1^.

**Figure 6:**
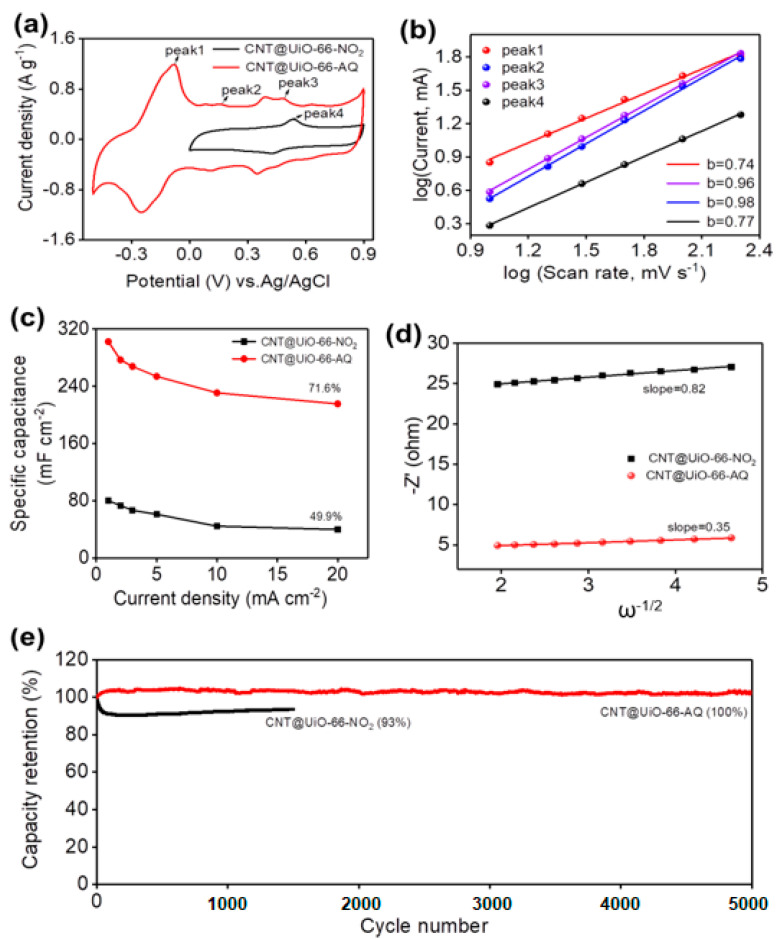
Electrochemical performance of CNT@UiO-66-NO_2_ and CNT@UiO-66-AQ in three-electrode electrochemical cells with 1 M H_2_SO_4_ aqueous electrolyte. (**a**) CV curves at a scan rate of 30 mV s^−1^. (**b**) The relationship between logarithm oxidation peak current and logarithm scan rate. (**c**) Variation of specific capacitance against the current densities. (**d**) Variations and fittings between Z_re_ and the reciprocal square root of the angular frequency in the low-frequency region of EIS plots. (**e**) Cycling performance of CNT@UiO-66-AQ at 5 mA cm^−2^.

For flexible energy storage, carboxylated CNTs-incorporated MOF hybrids are designed in our case as free-standing film electrodes, wherein the carboxyl groups serve as coordination sites for the metal nodes and anchor to improve the bonding strength between components in hybrids. The CV and GCD curves of CNT@UiO-66-NO_2_ and CNT@UiO-66-AQ (10 mV s^−1^) are shown in Figure 6a and Figure S8 (Supplementary Materials), respectively. As expected, CNT@UiO-66-AQ boosts significantly improved charge storage by taking advantage of both capacitance and redox reactions, when compared with CNT@UiO-66-NO_2_ or UiO-66-AQ. Three pairs of redox peaks can be observed in the CV curve of CNT@UiO-66-AQ. We thus explore the reaction kinetics through the equation *i* = aν^b^, where *i* and ν are the oxidation peak currents and scan rates. The b equal to 1 represents the capacitive process, while b of 0.5 refers to the redox process. In our cases, we obtain straight lines with the slope as b when plotting logarithm *i* against logarithm ν (Figure 6b). The b value is 0.74 for major peak 1 for CNT@UiO-66-AQ, suggesting a synergetic charge storage of capacitive and redox of AQ. The b values for peak 2 and 3 for CNT@UiO-66-AQ are quite close to 1, which is mainly attributed to the capacitance contribution of CNTs. Interestingly, the redox peak 4 accounting for ligands is marginal for UiO-66-NO_2_. CNT@UiO-66-AQ presents an excellent areal specific capacitance upto 302.3 mF cm^−2^ at 1 mA cm^−2^, which is nearly three-fold higher than that of CNT@UiO-66-NO_2_ (80.1 mF cm^−2^) (Figure S9, Supplementary Materials). By increasing the current density from 1 to 20 mA cm^−2^ (Figure 6c), CNT@UiO-66-AQ was found to retain 71.6% of its initial areal specific capacitance of (216.5 mF cm^−2^), outperforming CNT@UiO-66-NO_2_ with a capacitance retention rate of 49.9% under the same conditions. All the results reveal that the incorporation of redox pendants and CNTs substrate is beneficial, improving the conductivity, charge storage, and rate performance of hybrid electrodes simultaneously. 

**Figure 7:**
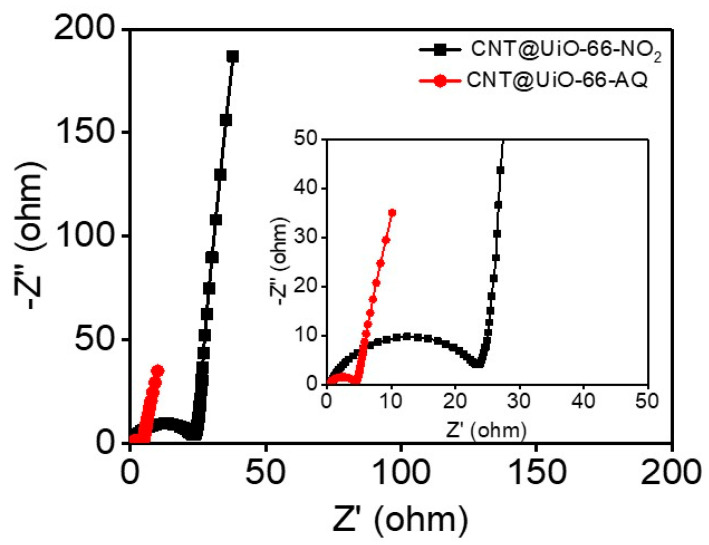
EIS plots of CNT@UiO-66-NO_2_ and CNT@UiO-66-AQ.

Electrochemical impedance spectroscopy (EIS) was used to reveal the charge and mass transport behavior of the electrodes. Generally, the intercept of the semicircle line at the x-axis represents the resistance (*R_s_*) of the electrolyte contacting with the current collector and electrode materials [37]. The charge transfer resistance (*R_ct_*) is calculated based on the diameter of the semicircle in the high-frequency region, while the straight line at low frequencies demonstrates the ion diffusion within electrode [38]. The steeper shape of the sloped line represents an ideal capacitive behavior with the faster diffusion of ions in electrolyte [39]. As expected, CNT@UiO-66-NO_2_ and CNT@UiO-66-AQ electrodes exhibit a straight line in the low-frequency region and a semicircle in the high-frequency region in the EIS spectra (Figure 7). CNT@UiO-66-AQ exhibits a dramatically lower *R_ct_* (4.4 Ω) than CNT@UiO-66-NO_2_ (23.4 Ω), which is consistent with their conductivity results. Importantly, both EIS spectra show almost vertical slopes in the low-frequency regions for two electrodes, indicating efficient ion diffusion.

In order to quantitatively evaluate the diffusion impedance, we further plot the real part of the collected impedance (Z′) versus the reciprocal square root of the sampling frequency (Figure 6d). From the slope (0.82 vs. 0.35) of as-obtained lines, we can conclude that CNT@UiO-66-AQ has much lower ion diffusion resistance than CNT@UiO-66-NO_2_ to promote more efficient mass transfer. This agrees well with ordered morphology and redox-assisted electrochemical kinetics for CNT@UiO-66-AQ electrodes (Figure 5e,f). Besides the superior capacitance and rate performance, CNT@UiO-66-AQ also shows good long-term electrochemical stability. As shown in Figure 6e, there was almost no drop in its initial specific capacitance after 5000 cycles, while only 93% retention of initial capacitance was obtained for CNT@UiO-66-NO_2_ after 1600 cycles. More importantly, during the continuous cycling of CNT@UiO-66-AQ, no AQ pendants cleavage or organic intermediate precipitation was observed, as traced by the color and composition of the electrolyte. This implies the excellent durability of azo linkage and its tolerance to strong acidic aqueous conditions. 

**Figure 8:**
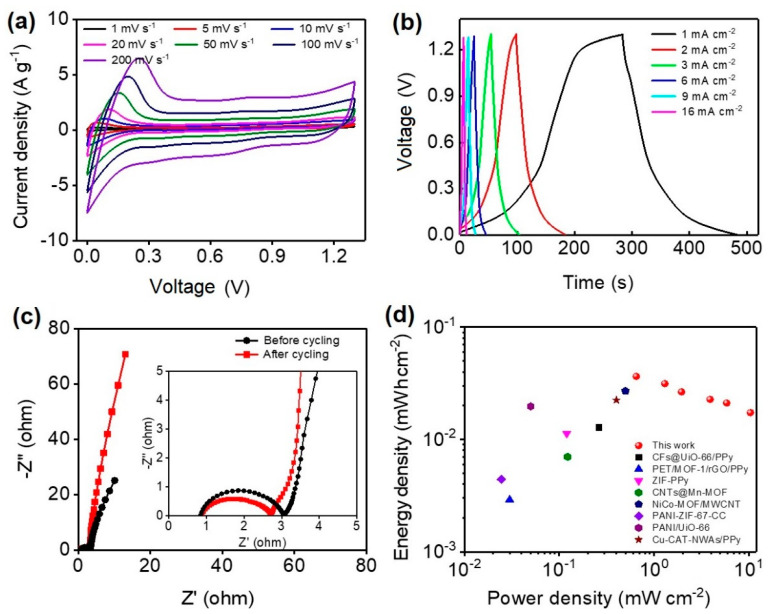
Electrochemical performance of CNT@UiO-66-AQ SSCs. (**a**) CV curves recorded at different scan rates. (**b**) GCD curves measured at different current densities. (**c**) EIS plots. (**d**) Ragone plots in comparison to representative high-performance MOF-based SSCs in the literature.

In order to evaluate the applicability of our designed CNT@UiO-66-AQ as a self-standing film electrode for flexible energy storage, we fabricated symmetrical supercapacitors (SSCs) by assembling two identical electrodes on both sides of a filter paper separator with 1 M H_2_SO_4_ as the electrolyte. Figure 8a shows the CV curves of an SSC at a scan rate that increased from 1 to 200 mV s^−1^. A pair of redox peaks can clearly be observed at operating voltage regions of 0~0.3 V, corresponding to the redox reactions of AQ moiety. According to the discharge curve, our SSC showcases an excellent areal capacitance of 155.4 mF cm^−2^ (1 mA cm^−2^). The capacitance can be retained by 48% when increasing the current density to 16 mA cm^−2^ (Figure 8b). The cyclic stability of the as-fabricated SSC device was revealed over 10,000 GCD cycles at 1 mA cm^−2^ (Figure S10, Supplementary Materials). During the cycling process, the specific capacitance declines by 20% after the first 4000 cycles before it slowly drops to 71.9% of the initial value at the end of 10,000 cycles. Such a high capacitance retention over such a long cycle life demonstrates the excellent cycling stability of our CNT@UiO-66-AQ-based SSCs. Figure 8c depicts the Nyquist plots of the initial cycle and after 10,000 cycles for the SSC. The longer Warburg line after long cycling implies sufficient electrolyte permeation and diffusion within the porous electrodes [40]. Meanwhile, the lower charge transport resistance (indicated by a smaller diameter of the semicircle) reveals the activation of the electrodes during cycling [41].

Power density (P) and energy density (E) are two key parameters used to evaluate the electrochemical performance for practical SC devices. Our SSC device can achieve a maximum P of 10.4 mW cm^−2^ and a highest E of 0.037 mWh cm^−2^ (Figure 8d), outperforming most MOFs-based SSCs in the literature. This can be attributed to the extended voltage window and improved specific capacitance of the CNT@UiO-66-AQ electrode. Representative device data (P, E) for the high-performance SSCs are listed as follows for comparison: NiCo-MOF/MWCNT (0.499 mW cm^−2^, 0.027 mWh cm^−2^) [39], CFs@UiO-66/PPy (0.26275 mW cm^−2^, 0.0128 mWh cm^−2^) [42], PET/MOF-1/rGO/PPy (0.03 mW cm^−2^, 0.0029 mWh cm^−2^) [43], ZIF-PPy (0.12 mW cm^−2^, 0.0113 mWh cm^−2^) [44], CNTs@Mn-MOF (0.12260 mW cm^−2^, 0.00699 mWh cm^−2^) [45], PANI-ZIF-67-CC (0.82 mW cm^−2^, 0.3396 mWh cm^−2^) [46], PANI/UiO-66 (0.05 mW cm^−2^, 0.0197 mWh cm^−2^) [47], Cu-CAT-NWAs/PPy (0.4 mW cm^−2^, 0.0224 mWh cm^−2^) [48]. In a word, the MOFs used in our electrode have played at least three roles: (i) providing the anchoring sites to bond with CNTs and AQ to improve both conductivity and energy storage capability, (ii) affording a highly porous framework to promote electrochemical dynamics, and (iii) separating CNTs and redox functionalities to allow different energy storage mechanisms to work for optimal performance.

## 3. Experimental Section

### 3.1. Synthesis of UiO-66-NO_2_

UiO-66-NO_2_ was synthesized according to the reported procedure with slight modification [15]. Briefly, ZrCl_4_ (233 mg), 2-nitroterepthalic acid (233 mg) and 3-aminobenzoic acid (244 mg) were added into DMF (20 mL) and dissolved by the addition of 0.16 mL of concentrated hydrochloric acid. The solution was then transferred into a Teflon-lined stainless-steel autoclave (40 mL) and heated at 120 °C for 2 days. After cooling to room temperature, the reaction mixture was filtered to wash the precipitate with DMF and methanol in sequence three times. The crude product was then stirred in methanol for three days. Further filtration and drying under vacuum at 120 °C for 12 h afforded the *title* product as a pale-yellow powder (190 mg, 65% yield) (inset photo in Figure 1b).

### 3.2. Synthesis of UiO-66-AQ 

The grafting of redox pendants onto nitro-functionalized MOF via azo linkage was realized using solvothermal reaction in the mixture solution of DMF and water. In our case, UiO-66-NO_2_ (100 mg) was first dispersed in DMF (10 mL), then 1-nitroanthraquinone (100 mg) and deionized water (30 mL) were added in sequence. The mixture was transferred into a Teflon-lined stainless-steel autoclave and heated at 180 °C for 12 h. The collected dark purple solid was washed several times using DMF until the filtrate became colorless. The crude product was stirred in methanol for three days, and the product (inset photo in Figure 1c) was obtained via filtration and dried under a vacuum at 120 °C for 12 h.

### 3.3. Preparation of Self-Standing CNT@UiO-66-NO_2_

Carboxylated single-walled CNTs (80 mg) were dispersed in DMF (16 mL) and sonicated for 30 min. Then, the MOF precursor solution [ZrCl_4_ (85 mg), 2-nitroterepthalic acid (85 mg), and 3-aminobenzoic acid (89 mg) mixed in DMF (8 mL) with concentrated hydrochloric acid (60 μL)] was added. The mixture was transferred into a Teflon-lined stainless-steel autoclave and heated at 120 °C for 2 days. The suspension was centrifugated three times using DMF. The obtained black powders were dispersed in ethanol and filtered into homogenous films (Figure 1d). A further freeze-thawing readily afforded a flexible and stretchable thin film (ca. 70 μm), which was cut into 1 cm × 1 cm size to use as self-standing electrodes.

### 3.4. Preparation of CNT@UiO-66-AQ Self-Standing Film Electrode

CNT@UiO-66-NO_2_ black powder was dispersed into DMF (10 mL), then 1-nitroanthraquinone (100 mg) and deionized water (30 mL) was added in sequence. After sonication for 30 min, the mixture was transferred into a Teflon-lined stainless-steel autoclave and heated to 180 °C for 12 h. The black powders were obtained after centrifugations by using DMF, dichloromethane, and methanol, respectively. The obtained black powders were dispersed in ethanol and filtered into homogenous films (ca. 40 μm) (Figure 1e). Similarly, the flexible CNT@UiO-66-AQ self-standing film electrodes were prepared via freeze-thawing and cut procedures.

### 3.5. Preparation of Reference Electrode 

Electroactive powder materials (UiO-66-NO_2_ and UiO-66-AQ) were mixed with acetylene black and poly(tetrafluoroethylene) with a weight ratio of 8:1:1. The as-obtained paste was pressed onto carbon cloth. The as-prepared composite membrane was cut into square pieces with 1 × 1 cm size and used directly.

### 3.6. Assembly of Symmetric Supercapacitors (SSCs)

The coin-cell shape SSCs were fabricated by sandwiching two electrodes with one filter paper as the separator, where aqueous 1 M H_2_SO_4_ was used as the electrolytes.

## 4. Conclusions

In summary, we have successfully designed redox MOFs incorporating CNTs hybrid for high-performance and cycling-stable electrodes for flexible energy storage. The azo-coupling AQ onto UiO-66 MOFs was realized via facile solvothermal reaction. The growth and morphology of crystalline MOFs was finely controlled in the presence of CNTs. The hybrid showcases a three-dimensional interpenetrating network, affording improved porosity and electric conductivity. The as-designed CNT@UiO-66-AQ electrode offers an extended potential window (−0.4~1 V), excellent energy storage (to 302.3 mF cm^−2^ at 1 mA cm^−2^), and negligible loss of capacitance over 5000 cycles in aqueous acidic electrolyte. Electrochemical kinetics reveal the interplay of capacitance and redox reaction, accompanying efficient charge and ion transfer. The symmetrical supercapacitor assembled by two CNT@UiO-66-AQ self-standing film electrodes deliver a maximum energy density of 0.037 mW h cm^−2^ and a highest power density of 10.4 mW cm^−2^, together with 71.9% capacity retention after 10,000 GCD cycles. Such device performance, to the best of our knowledge, is superior to most analogue MOFs electrode-based SSCs. Our post-synthetic azo-coupling redox pendants approach can be extended to different MOFs skeletons and redox nitroaromatics for the design of high-performance flexible energy storage.

## Supplementary Materials

The following Supplementary Materials can be downloaded at: https://www.mdpi.com/article/10.3390/molecules28227479/s1.
