# Supplementary material for: Azo-Linkage Redox Metal–Organic Framework Incorporating Carbon Nanotubes for High-Performance Aqueous Energy Storage
Source: Molecules. 2023 Nov 8;28(22):7479. doi: 10.3390/molecules28227479 (PMC10673354; doi:10.3390/molecules28227479)
Supplement: Supplementary file 1 [file molecules-28-07479-s001.zip › molecules-2648320-supplementary.pdf]

Supporting Information

Azo-Linkage Redox Metal–Organic Framework Incorporating Carbon Nanotubes for High-Performance Aqueous Energy Storage

Hualei Zhang [1,2], Xinlei Wang [1,2], Jie Zhou [2], Weihua Tang [1,2]

College of Materials, Xiamen University, Xiamen 361005, China

School of Chemistry and Chemical Engineering, Nanjing University of Science and Technology, Nanjing 210094, China

Table of content

1. Chemicals and materials……………………..…….….…….…………………2

2. Materials characterizations……………………….….……..…………………2

3. Electrochemical measurements……………………..…………………............3

4. FT-IR and XPS spectra……………………………………………..…………4

5. SEM and elemental mapping for MOFs……………………………...…………5

6. HRTEM, HAADF and elemental mapping for CNTs@MOFs................................6

7. TGA analysis...6

8. CV and GCD curves for MOFs and their hybrids with CNTs……………..………7

9. Cycling performance of SSC……………………………………….………..8

1. Chemicals and materials

N,N-dimethylformamide (DMF) was purchased from Energy Chemical Co., Ltd. Carboxylated single-walled carbon nanotubes (SWCNTs) (1~2 nm in diameter, 5~30 μm in length) were obtained from XFNANO Materials Tech Co. Ltd. Zirconium (IV) tetrachloride, 3-aminobenzoic acid, 2-nitroterephthalic acid and 1-nitroanthraquinone were purchased from Shanghai Macklin Biochemical Co., Ltd. All the chemicals were used directly without further purification.

2. Materials characterizations

X-ray diffraction (XRD) patterns were recorded on Bruker D8 Advance X-ray diffractometer using Cu Ka radiation (λ≈1.54 Å) at 40 kV and 40 mA. Fourier Transform Infrared (FT-IR) transmission spectra were collected from a BRUKER-EQUINOX-55 IR spectrophotometer. Scanning electron microscopy (SEM) was performed on a FEI quanta 400 FEG. Transmission electron microscopy (TEM) was performed on a JEM F2100F. High resolution transmission electron microscopy (HR-TEM) was performed on a FEI Talos F200S. The thermogravimetric analysis (TGA) was operated on TA Instruments SDTA851e with the heating rate of 10°C min^{-1} in the range 50-800 °C under nitrogen atmosphere. The electric conductivity data was obtained from a RTS-8 four-probe tester. X-Ray photoelectron spectroscopy (XPS) measurements were performed with a Thermo ESCALAB 250 X-ray photonelectron spectrometer using Al Kα radiation (hν ≈1486.7 eV) as the excitation source. Prior to the BET measurements, the samples were outgassed for 10 hours at 100 °C.

Adsorption isotherms were calculated for nitrogen adsorption at 77 K and pressures up to 1 bar, measured by Brunauer-Emmet-Teller (BET) surface analyzer (Micromeritics, ASAP 2020).

3. Electrochemical measurements

All the electrochemical measurements were carried out on a CHI760D electrochemical workstation (Shanghai, China). The electrochemical performance of as-prepared electrodes was first evaluated in a three-electrode cell using aqueous 1 M H_2SO_4 electrolyte, platinum foil as the counter electrode and Ag/AgCl as the reference electrode. The working electrodes were prepared by mixing active material (UiO-66-NO_2 or UiO-66-AQ), acetylene black, and poly(tetrafiuoroethylene) (PTFE) with a weight ratio of 80:10:10 and pressing onto carbon cloth as the current collector. The as-prepared composite membrane with 1 cm × 1 cm size was used as self-standing working electrode.

The areal capacitance (C_A) was calculated from GCD curves using the following equation: $C_A = I \times \Delta t / (S \times \Delta V)$ (1)

where I is the current density, Δt is the discharge time, ΔV is the voltage range, and S is the area of the entire electrode.

Energy density (E) and power density (P) of the symmetrical supercapacitor are calculated according to the following equations:

$$E = C_A \times (\Delta V)^2/(2\times3600) \quad (2)$$

$$P = E \times 3600/\Delta t \quad (3)$$

where C_A is the areal capacitance, ΔV is the voltage window and Δt is the discharge time.

4. FT-IR and XPS spectra

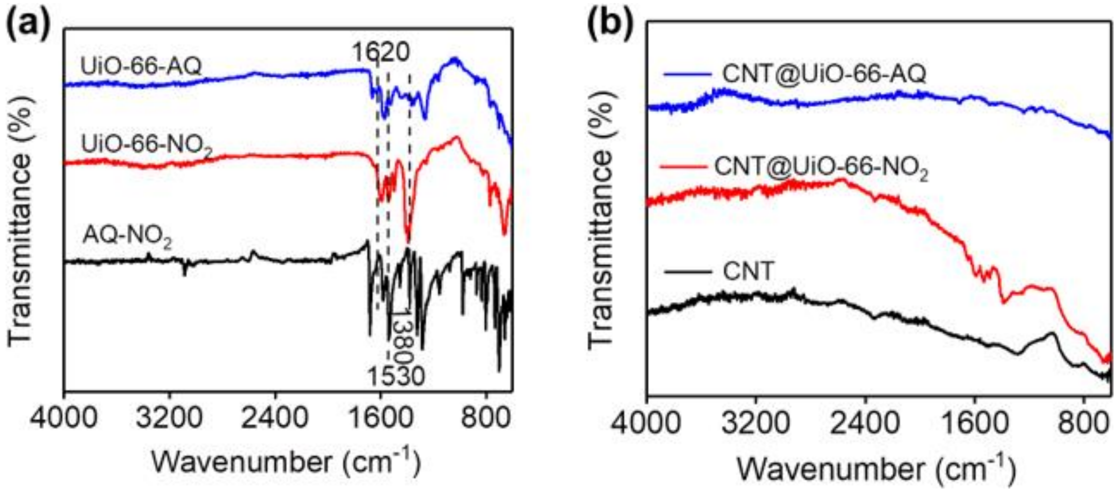

Figure S1. (a) FT-IR spectra of AQ-NO_2 and MOFs. (b) FT-IR spectra of CNT, CNT@UiO-66-NO_2 and CNT@UiO-66-AQ.

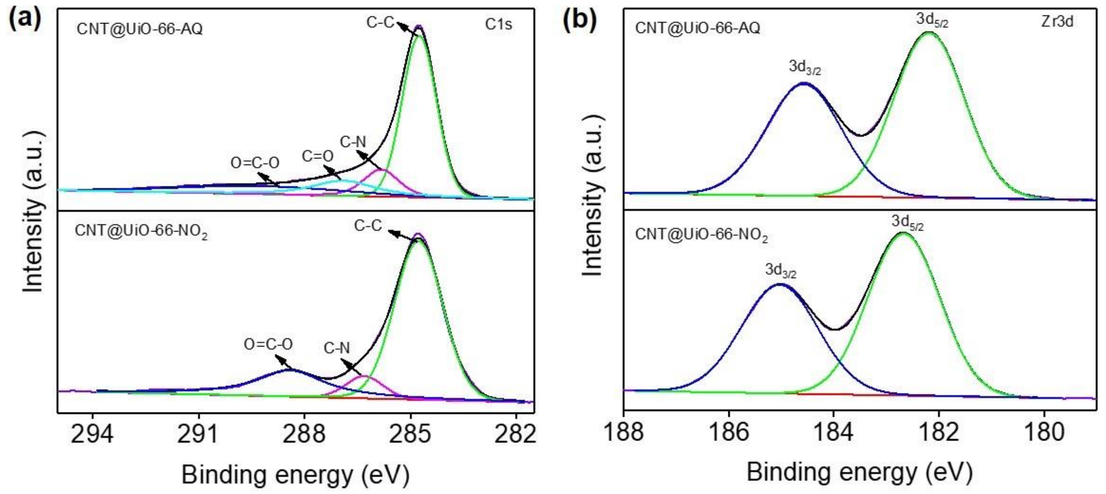

Figure S2. The comparison of (a) C 1s spectra and (b) Zr 3d spectra for CNT@UiO-66-NO_2 and CNT@ UiO-66-AQ.

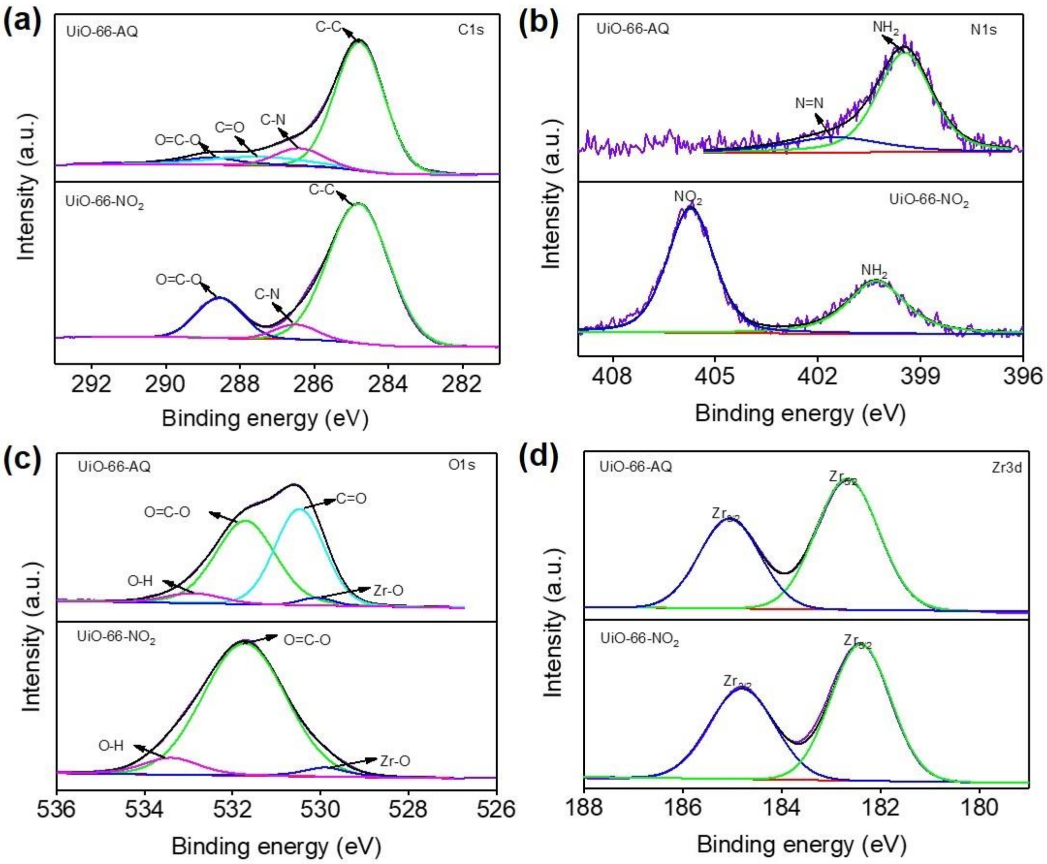

Figure S3. High-resolution XPS spectra of (a) C 1s, (b) N 1s, (c) O 1s and (d) Zr 3d for UiO-66-NO_2 and UiO-66-AQ powders.

5. SEM and elemental mapping for MOFs

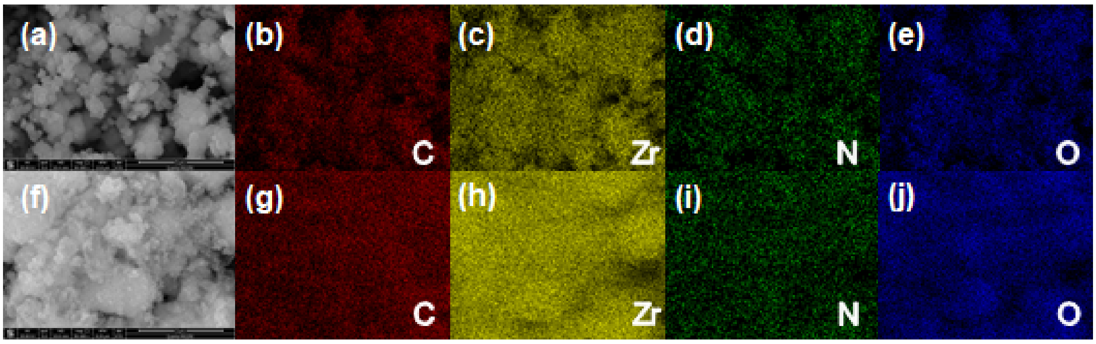

Figure S4. SEM image and the corresponding elemental (ca. C, Zr, N, and O) mapping images for (a-e) UiO-66-NO_2 and (f-j) UiO-66-AQ.

6. HRTEM, HAADF and elemental mapping for CNTs@MOFs

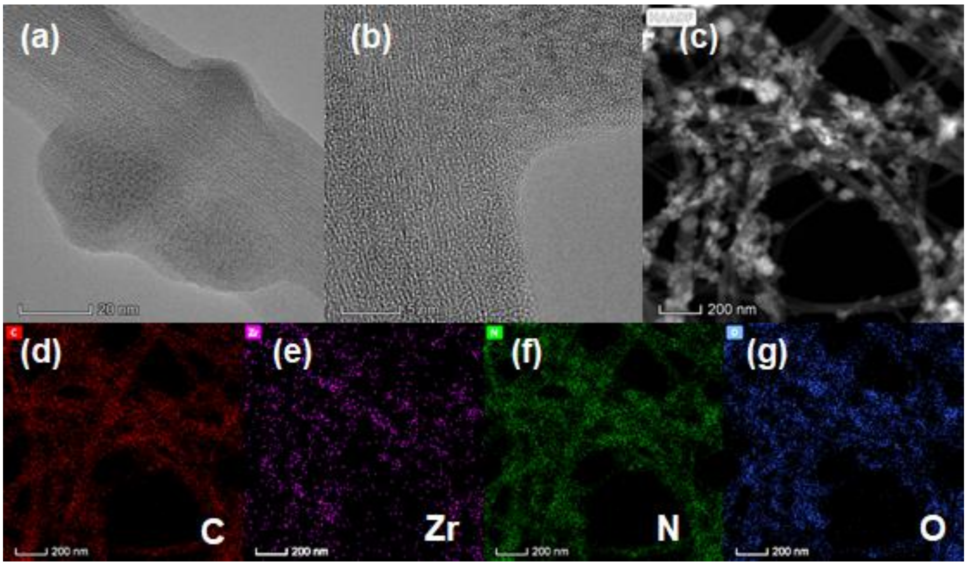

Figure S5. HRTEM images of (a,b) CNT@UiO-66-NO_2. (e) HAADF image of CNT@UiO-66-NO_2 and (d-g) corresponding element mapping of C, Zr, N and O, respectively.

7. TGA analysis

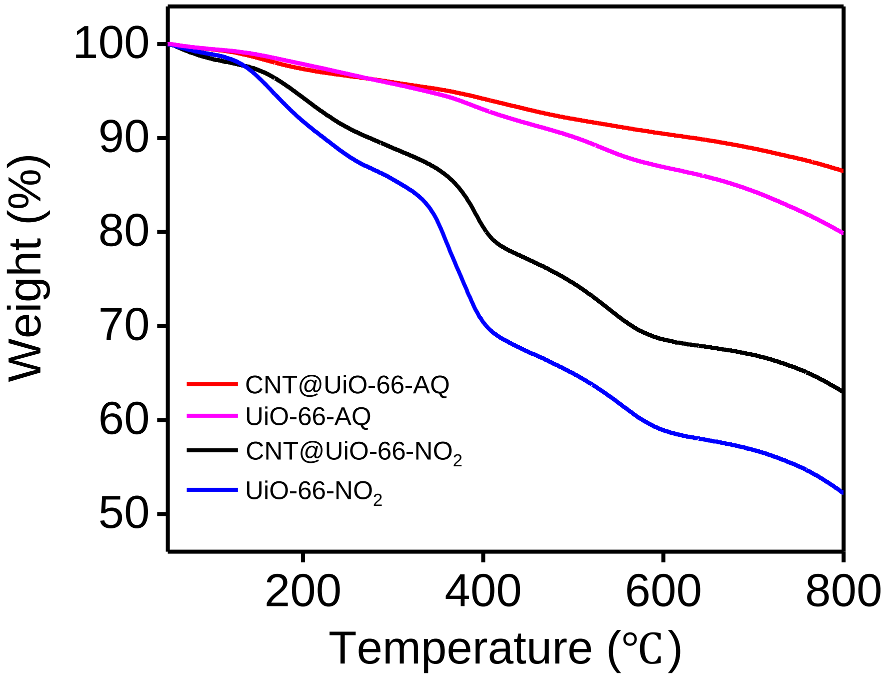

Figure S6. Comparison of TGA curves for MOFs and their hybrids with CNTs.

8. CV and GCD curves for MOFs and their hybrids with CNTs

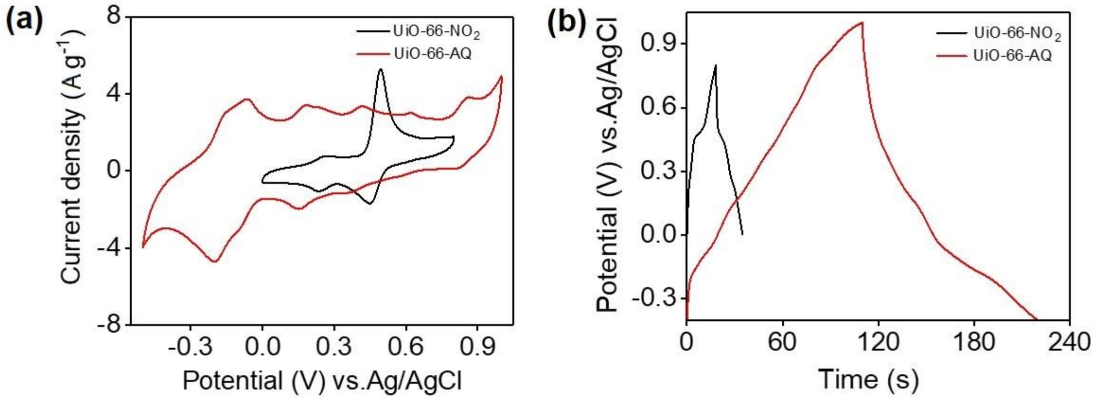

Figure S7. Electrochemical performance of powder electrodes UiO-66-NO_2 and UiO-66-AQ powders in a three-electrode configuration using 1 M H_2SO_4 aqueous solution as electrolyte. (a) Cyclic voltammetry (CV) curves at the same scan rate of 30 mV s^{-1}. (b) Galvanostatic charge/discharge (GCD) curves at a current density of 0.5 A g^{-1}.

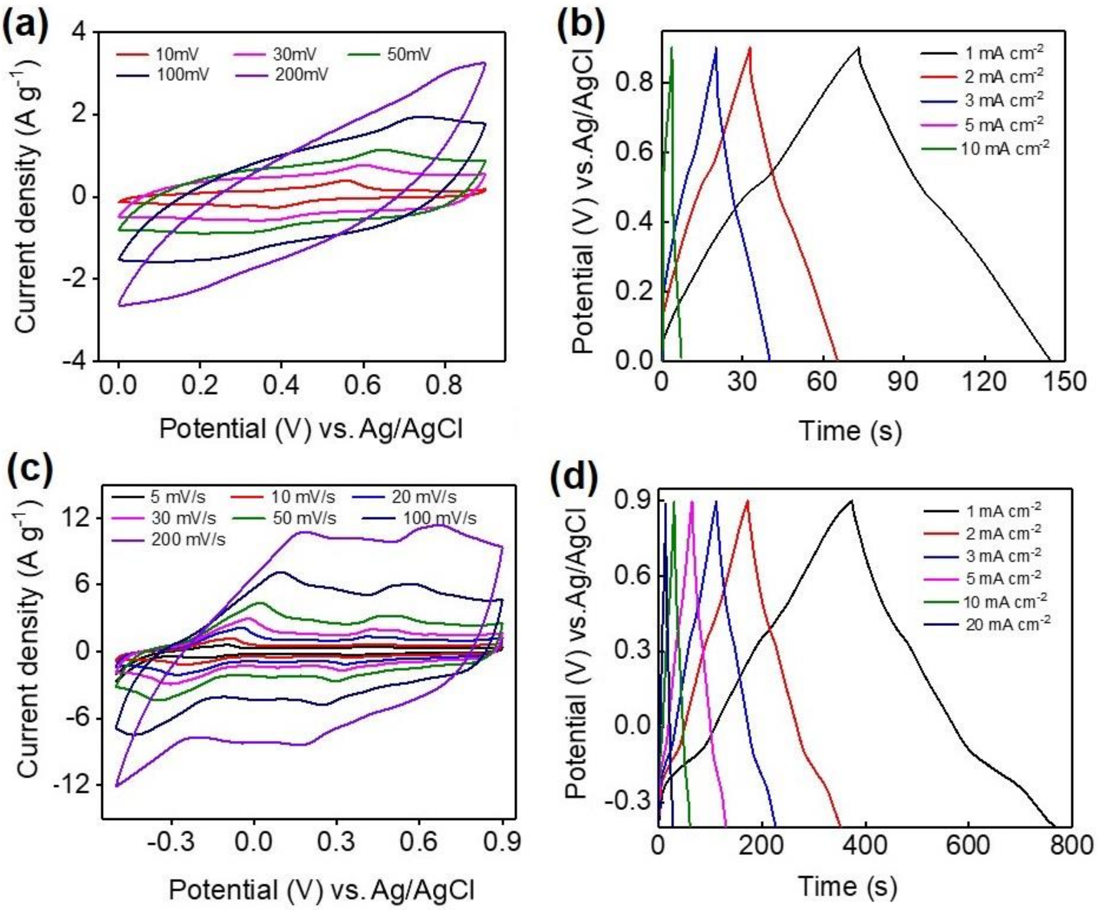

Figure S8. (a) CV curves and (b) GCD curves of CNT@UiO-66-NO_2. (c) CV curves and (d) GCD curves of CNT@UiO-66-AQ.

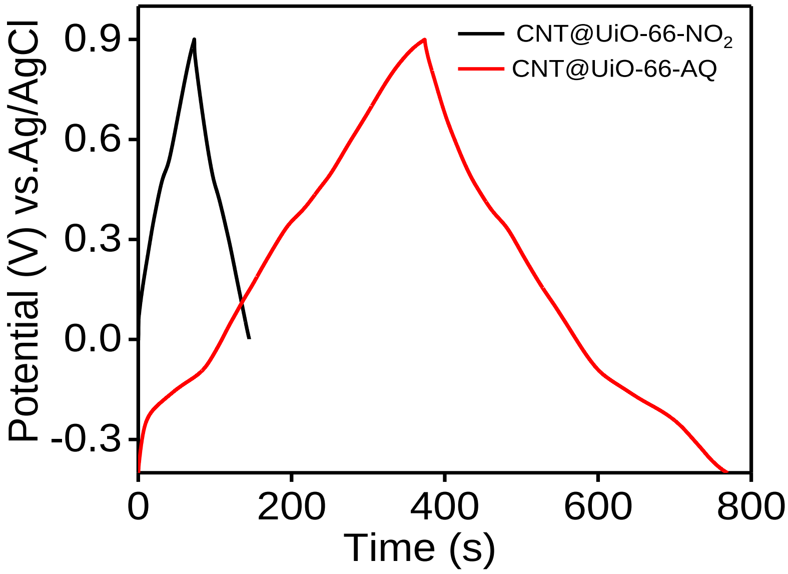

Figure S9. GCD curves of CNT@UiO-66-NO_2 and CNT@UiO-66-AQ at 1 mA cm^{-2}.

9. Cycling performance of SSC

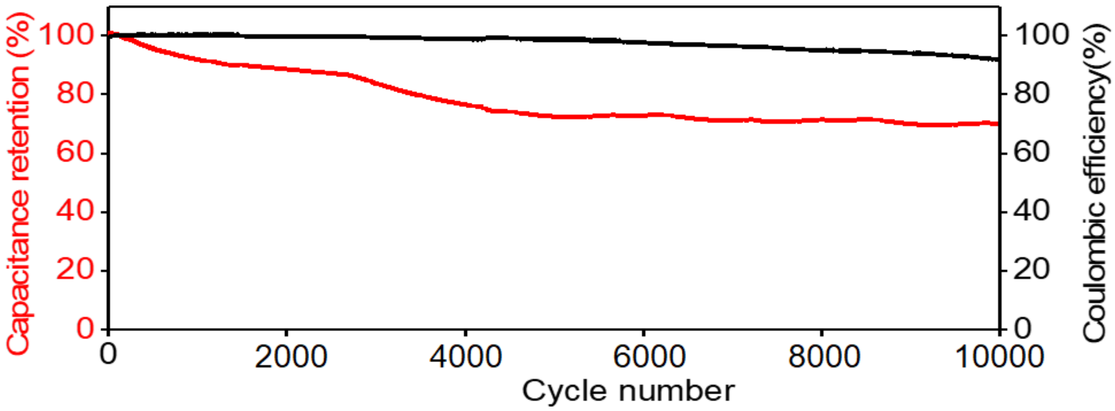

Figure S10. Cycling performance of CNT@UiO-66-AQ symmetric supercapacitor measured at 5 mA cm^{-2} over 10000 cycles with corresponding coulombic efficiency.

This can be attributed to the extended voltage window and improved specific capacitance of CNT@UiO-66-AQ electrode. Representative device data (P, E) for the high-performance SSCs are listed as follows for comparison: NiCo-MOF/MWCNT (0.499 mW cm^{-2}, 0.027 mWh cm^{-2}) [39], CFs@UiO-66/PPy (0.26275 mW cm^{-2}, 0.0128 mWh cm^{-2}) [42], PET/MOF-1/rGO/PPy (0.03 mW cm^{-2}, 0.0029 mWh cm^{-2}) [43] , ZIF-PPy (0.12 mW cm^{-2}, 0.0113 mWh cm^{-2}) [44], CNTs@Mn-MOF (0.12260 mW cm^{-2}, 0.00699 mWh cm^{-2}) [45], PANI-ZIF-67-CC (0.82 mW cm^{-2}, 0.3396 mWh cm^{-2}) [46], PANI/UiO-66 (0.05 mW cm^{-2}, 0.0197 mWh cm^{-2}) [47], Cu-CAT-NWAs/PPy (0.4 mW cm^{-2}, 0.0224 mWh cm^{-2}) [48].
